# The psychological burden experienced by Chinese citizens during the COVID-19 outbreak: prevalence and determinants

**DOI:** 10.1186/s12889-020-09723-0

**Published:** 2020-10-27

**Authors:** Zhengjia Ren, Yuchu Zhou, Yanhong Liu

**Affiliations:** 1 Department of Clinical Psychology, The Third Affiliated Hospital of Chongqing Medical University, Chongqing, 401120 China; 2 Daka Education Consulting Co.Ltd., Chengdu, Sichuan China; 3 Department of psychology, Hebei University of Chinese Medicine, Shijiazhuang, Hebei China

**Keywords:** Depression, Anxiety, COVID-19, Risk factors, Prevalence

## Abstract

**Background:**

The present study is aims to investigate the prevalence and determinants of depression and anxiety among the general population in the context of coronavirus disease 2019 (COVID-19) outbreak in China.

**Methods:**

A cross-sectional self-report survey methodology was used to gather the following data from Chinese citizens: sociodemographic information, physical and mental health disorder history, daily online time, social media exposure, feeling toward social media exposure, perception of the disease, infection cases in the local area, and previous experiences with stressful life incidents. Levels of anxiety and depression were self-reported employing the Generalized Anxiety Disorder 7-item scale and the Patient Health Questionnaire 9-item scale, respectively.

**Results:**

Among the 6130 participants, the prevalence of anxiety and depression was 7.1 and 12%, respectively. Multiple logistic regression analysis revealed that psychological disturbances were associated with gender, people with religious background, being a medical professional, having physical or mental health disease, difficulty accessing medical aids, experience with traumatic incidents, the perceived possibility of sequelae after being cured of COVID-19, daily online time, the source of the information relevant to COVID-19, frequency of receiving information regarding COVID-19, and negative feelings triggered by social media.

**Conclusions:**

There needs to be a consistent message from authorities to reduce the panic and confusion of the public, and to decrease public exposure to persistently negative information. It is necessary to help people transform their negative experiences into positive changes especially for individuals with physical illness, individuals with mental health disorders, and medical professionals.

**Supplementary Information:**

The online version contains supplementary material available at 10.1186/s12889-020-09723-0.

## Background

In 2019, China experienced an pandemic of COVID-19 at the end of 2019. The impact of COVID-19 was enormous and widespread, and it threatened psychosocial welling, economic stability, and normal daily life in the public. Studies of the psychological effect of COVID-19 at a general population level have been reported. Wang al. (2020) found that approximately 28.8% of respondents reported significant anxiety symptoms, and 16.5% of respondents reported moderate to severe depressive symptoms during the COVID-19 pandemic [1]. Another study reported that approximately 8.1, 28.8, and 16.5% of participants reported clinical symptoms of stress, anxiety, and depression, respectively, and there were no significant changes after 4 weeks of follow-up [2].

According to stress-vulnerability model, emotional problems in stressful situations are related to an individual’s vulnerability to stress, the amount of stress, the influence of the environment, and the individual’s ability to cope with stress [3]. Previous studies have found that people who need to stay in their jobs during the pandemic face double stressors - the stress of overwork load and the risk of infection, which make them susceptible to psychological disturbance such as medical personnel [4, 5]. In addition, people with mental illness have higher levels of anxiety, depression and even suicidal ideation than the general population [6]. Thus, the impact of COVID-19 on the mental health of the different population is prevail and widespread.

In order to provide effective intervention, an increasing number of studies have attempted to determine the potential toxic stressors related to mental disorders among the public, such as anxiety and depression [7]. There has been an increase in outbreak across provinces and countries, and the unpredictable future of this pandemic has been exacerbated by myths and misinformation from the internet, thus inducing anxiety, panic, and desperation in the population [8, 9]. In China, the number of Chinese internet users is 829 million, and an increasing number of individuals are using smartphones as their primary mode of internet access to obtain information, thus increasing opportunities for information dissemination and increasing the potential impact on the population [10]. Obviously, increased exposure to negative news from social media could make it increasingly difficult for people to distinguish fact from fiction, which is a toxic stressor to individual mental health.

Previously reported predictors of psychiatric complications during communicable disease outbreaks include sociodemographic variables [e.g., being a health care worker (HCW)] [4, 5], severe outbreaks of infectious diseases locally [11], health beliefs toward infectious diseases [e.g., perceived risk or threat of infection] [12, 13], and psychosocial variables [e.g., social support, medical history of mental health disorders and physical diseases] [14]. In addition, researchers and clinicians have stated that past experiences toward life adversities could influence an individual’s current response to life incidents, such that individuals who had positive experiences toward life adversities were more likely to engage in positive coping or attitudes toward uncertainty in life, and vice versa [15]. Risk factor analysis can improve the detection of hidden psychiatric complications. The present study aimed to identify the prevalence and determinants of anxiety and depression during the COVID-19 outbreak. This research can lead to effective preventive strategies and recommendations for preventing mental health problems among the general population.

## Methods

### Participants

The approval of the study was given by the institutional review committee of Hebei University of Chinese Medicine. We used a convenience sampling method. For this cross-sectional study, all participants were directly recruited online from February 9 to February 20. The participants age above 16 years old who is currently in the mainland of China were invited to complete the questionnaires through an online questionnaire platform called Wenjuanxing. If the participants consented to participate in the study, they completed the questionnaires anonymously. They could stop or quit the study at any time without facing any repercussions. Due to the recruitment methodology, a response rate could not be calculated. A total of 6130 participants completed the questionnaires.

### Measures

#### Demographic information

A short demographic questionnaire was developed to gather information from the participants, namely, their age, gender, marital status, income, career, national ethnic minority (yes or no), and religion.

### The COVID-19 outbreak situation in their city

One item asked the respondent to report the number of confirmed infections in their city (0–9, 10–30, 31–100, 101–399, 400 or above).

#### Perceived belief toward COVID-19

Each participant responded to the following items on a scale from 1 (very low chance) to 10 (very high chances): the likelihood that you think your family member will be infected; the likelihood that you will be infected; the likelihood that colleagues and friends will be infected; the likelihood that this disease will be cured; the likelihood that a person who is cured of the disease will have sequelae.

#### The information relevant to online time

Participants responded to two items. What is the average amount of time you spend online daily (0–3 h; 3–5 h; 5–7 h; 7–9 h; more than 9 h)? (2) How often do you receive new information about the coronavirus each day (rarely, sometimes, often, all the time)?

#### The source of the information relevant to COVID-19

Participants reported whether they received information from any of the following channels (yes or no): (1) newspaper, (2) mobile news client, (3) searching the internet by oneself, (4) WeChat group, (5) state media on television, (6) messages from friends, (7) messages from family, and (8) community propaganda.

#### Medical history

Participants responded to the following items regarding their medical history. Do you have any chronic physical disease that requires regular medical attention (yes or no)? Please select the disease you have been diagnosed from the list below such as depression, anxiety, bipolar disorder, schizophrenia, obsessive disorder, or psychosomatic disorder.

#### Difficulty in seeking medical treatment

Participants responded to the following items regarding their medical treatment. Have you had any difficulty seeking medical treatment recently (did not seek medical advice, not difficult, or difficult)?

#### The influence of past life events

Participants responded to the following items regarding their past life events. Have you experienced any significant life events (natural and man-made diseases, etc.)? To what extent have they affected you (negative influence on my life, positive influence on my life, I did not experience any significant life events)?

#### The extent feeling to which you have received information about the COVID-19

Participants reported their feelings of desperation, fear, confusion, anger, sadness, and somatic discomfort on a scale from 1 (not at all) to 10 (very strong).

### Psychosocial measures

#### Generalized anxiety disorder 7-item (GAD-7) scale

A Chinese version of GAD-7 was applied to evaluate generalized anxiety symptoms in the past 2 weeks, the participants were required to rate on a 4-point scale from 0 (not at all) to 3 (nearly every day) [16]. GAD-7 scores from 0 to 21; 5, 10 and 15 are the thresholds for mild, moderate and severe anxiety symptoms, respectively. Higher scores indicate more symptoms. The measure of this study reflected adequate internal consistency with Cronbach’s alpha = .95. A cutoff point of 10 was set, and those who had scores of 10 and above were considered to likely have anxiety. The current study defined parents with a score above 9 as having anxiety symptoms (GAD-7 score >  9).

#### Patient health questionnaire 9-item (PHQ-9) scale

Depression was assessed by using a Chinese version PHQ-9. The PHQ-9 is a continuous assess of the frequency of depression symptoms in the past 2 weeks [17]. All the items were rated on a scale from 0 (not at all) to 3 (nearly every day). The sum score of PHQ-9 ranges from 0 to 27, severity is classified into five categories: minimal (0–4), mild (5–9), moderate (10–14), moderate (15–19) and severe (20–27). The validity as well as the reliability of the PHQ-9 have consistently been verified in different studies of the Chinese population. In the current research, the Cronbach’s alpha of PHQ-9 was 0.95. A cutoff point of 9 was set, and those who had scores of 9 above were considered to likely have depression. The current study defined parents whose score was above 9 as having depressive symptoms (PHQ-9 score >  9).

### Statistical analyses

Descriptive analysis of all demographic and study variables was processed by SPSS version 22.0. Binary logistic regression models were applied to identify factors of which influenced depression or anxiety. The level of depression or anxiety (i.e., at least moderate vs. below moderate) was used as the dependent variable, while sociodemographic data, physical or mental health disorder history, online time every day, perceived beliefs about COVID-19, social media exposure, feelings about social media exposure, and past experiences with stressful life events were used as independent variables. Multivariate models were set up by forward selection (likelihood ratio) method, and *p*-values of less than 0.05 were considered statistically significant.

## Results

### Prevalence of depression and anxiety

Overall, 12% (736/6130) of the participants experienced moderate or above depression symptoms, and 7.1% (437/6130) showed moderate or above symptoms of anxiety.

### Demographic characteristics

Of the 6130 participants who were included, 33.1% (2030/6130) were male, 67.9% (3351/6130) were single, 30.4% (1866/6130) were married or cohabitating, and 1.6% (101/6130) were divorced or other. The other characteristics of the participants are listed in Table 1.

**Table 1:** Demographics of the participants

Participants		*N* = 6130
Gender	Men	2030 (33.1)
	Women	4100 (66.9)
Age	16 < Age ≤ 20	1833 (29.9)
	21 < Age ≤ 30	2538 (41.4)
	31 < Age ≤ 40	800 (13.1)
	41 < Age ≤ 50	675 (11)
	Above 51	284 (4.6)
Career	Student	3415 (55.7)
	Civil servant	217 (3.5)
	Middle and senior manager	303 (4.9)
	blue-and-white collar workers	1232 (20.1)
	Professional staff	503 (8.2)
	medical professionals	460 (7.5)
Income (RMB)	Below 1000Yuan	3120 (50.9)
	1001–3000 Yuan	700 (11.4)
	3001–5000 Yuan	769 (12.5)
	5001–7000 Yuan	611 (10)
	7001–10,000 Yuan	442 (7.2)
	10,001 RMB Yuan	488 (8)
Marital status	Single	4163 (67.9)
	Married or cohabit	1866 (30.4)
	Divorced or other	101 (1.6)
Chronic physical disease	No	5674 (92.6)
	Yes	456 (7.4)
Religion	No	5503 (89.8)
	Yes	627 (10.2)
National ethnic minority	No	5778 (94.3)
	Yes	352 (5.7)
Education	Middle school and below	470 (7.7)
	Associate degree	638 (10.4)
	university	4381 (71.5)
	Master’s degree or above	641 (10.5)
Daily online time	0 h < time ≤ 3 h	781 (12.7)
	3 h < time ≤ 5 h	1825 (29.8)
	5 h < time ≤ 7 h	1510 (24.6)
	7 h < time ≤ 9 h	903 (14.7)
	> 9 h	1111 (18.1)
Frequency of receiving information regarding COVID-19	Rarely	276 (4.5)
	Sometimes	550 (9)
	often	3642 (59.4)
	All the time	1662 (27.1)
Difficulty accessing medical treatment	Not sick or not difficult	5840 (95.3)
	Yes	290 (4.7)
Experience with traumatic incidents	Negative	718 (11.7)
	Positive	1452 (23.7)
	Did not experiences	3961 (64.5)
Mental health disorder	None	4513 (73.7)
	Having one mental health disorder	992 (16.2)
	Comorbidities with one mental health disorders	407 (6.6)
	Comorbidities with two or more mental health disorders	218 (3.6)
Infection cases in local area	0–9	1361 (22.2)
	10–30	1960 (32)
	31–100	995 (16.2)
	101–300	1033 (16.9)
	301 above	781 (12.7)
COVID-19 information from newspaper	No	5695 (92.9)
	Yes	435 (7.1)
COVID-19 Information from News App	No	1433 (23.4)
	Yes	4697 (76.6)
Self-searching COVID-19 information	No	2632 (42.9)
	Yes	3498 (57.1)
COVID-19 information from WeChat group	No	2433 (39.7)
	Yes	3697 (60.3)
COVID-19 information from state media on TV	No	2355 (38.4)
	Yes	3775 (61.6)
COVID-19 information from friends	No	5037 (82.2)
	Yes	1093 (17.8)
Message from family	No	4324 (70.5)
	Yes	1806 (29.5)
Publicity from community worker	No	4446 (72.5)
	Yes	1684 (27.5)
Desperation induced by information relevant to COVID-19	M ± SD	1.91 (1.77)
Fear induced by information relevant to COVID-19	M ± SD	4.01(±2.64)
Confusion induced by information relevant to COVID-19	M ± SD	3.55(±2.65)
Anger induced by information relevant to COVID-19	M ± SD	3.87(±3.06)
Sadness induced by information relevant to COVID-19	M ± SD	3.86(±2.88)
Somatic discomfort induced by information relevant to COVID-19	M ± SD	1.94 (±1.73)
The likelihood that oneself get infected	M ± SD	1.94(±1.73)
The likelihood that your family member get infected	M ± SD	1.96(±1.73)
The likelihood of having sequela after cured	M ± SD	4.65(±2.85)
The likelihood that this disease will be cured	M ± SD	6.7 (±2.87)

The official exchange rate was approximately US$ = 6.99Yuan. COVID-19: novel coronavirus (2019-nCoV)–infected pneumonia

### Factors associated with depression and anxiety (Tables 2 and 3)

The multivariate logistic regression analysis controlling for the simultaneous association of risk factors indicated that a previously diagnosed mental health disorder (OR = 2.294, 95% CI = 1.847–2.850, *P* < 0.000), a previously diagnosed mental health disorder comorbid with one mental illness (OR = 3.525, 95% CI = 2.683–4.631, *P <* 0.000), a previously diagnosed mental health disorder comorbid with two or more mental illnesses (OR = 5.897, 95% CI = 2.683–4.631, *P <* 0.000), perceived extent of possible sequelae after treatment for COVID-19 (OR = 1.046, CI = 1.02–1.04, *p* = 0.006), desperation induced by information relevant to COVID-19 (OR = 1.128, CI =1.074–1.185, *p <* 0.000), confusion induced by information relevant to COVID-19 (OR = 1.088, CI =1.044–1.134, *p <* 0.000), sadness induced by information relevant to COVID-19 (OR = 1.054, CI =1.014–1.096, *p* = 0.008), and other related factors were significant in the multivariate logistic regression model were summarized Table 2,thus indicating their relevance as important related factors for depression among the population

**Table 2:** Logistic regression analysis of the influencing factors of depression among population

		P	OR	95% C.I.
Gender	Women			
	Men	0.000	1.433	1.186–1.732
Age	16 < Age ≤ 20	0.003		
	21 < Age ≤ 30	0.104	1.194	0.964–1.477
	31 < Age ≤ 40	0.265	0.841	0.621–1.140
	41 < Age ≤ 50	0.068	0.719	0.504–1.025
	16 < Age ≤ 20	0.078	0.608	0.350–1.057
The likelihood of having sequela after cured	Mean ± SD(4.65 ± 2.85)	0.006	1.046	1.013–1.081
Self-searching COVID-19 information	No			
	Yes	0.041	0.828	0.691–0.992
	No			
COVID-19 information from state media on TV	Yes	0.031	0.821	0.687–0.982
	No			
Chronic physical disease	Yes	0.034	1.386	1.025–1.873
Desperation induced by information relevant to COVID-19	Mean ± SD(1.91 ± 1.77)	0.000	1.128	1.074–1.185
Confusion induced by information relevant to COVID-19	Mean ± SD (3.55 ± 2.65)	0.000	1.088	1.044–1.134
Sadness induced by information relevant to COVID-19	Mean ± SD (3.86 ± 2.88)	0.008	1.054	1.014–1.096
Somatic discomfort induced by information relevant to COVID-19	Mean ± SD (1.94 ± 1.73)	0.000	1.185	1.130–1.242
Mental health disorder	None	0.000		
	Having one mental health disorder	0.000	2.294	1.847–2.850
	Comorbidities with one mental health disorders	0.000	3.525	2.683–4.631
	Comorbidities with two or more mental health disorders	0.000	5.897	4.177–8.324
Daily online time	0 h < time ≤ 3 h	0.000		
	3 h < time ≤ 5 h	0.269	1.216	0.860–1.719
	5 h < time ≤ 7 h	0.651	1.086	0.759–1.554
	7 h < time ≤ 9 h	0.000	2.034	1.412–2.930
	> 9 h	0.000	2.567	1.816–3.628
Experience with traumatic incidents	Negative	0.000		
	Positive	0.000	0.434	0.331–0.569
	Did not experiences	0.000	0.517	0.410–0.653
Constant		0.000	0.017

**Table 3:** Logistic regression analysis of the influencing factors of anxiety among population

		Sig.	OR	95% C.I.
Gender	Women	Reference		
	Men	0.018	1.359	1.054–1.752
career	Civil servant	0.007		
	Student	0.754	1.114	0.568–2.185
	Middle and senior manager	0.310	1.511	0.681–3.354
	blue-and-white collar workers	0.298	1.437	0.726–2.844
	Professional staff	0.744	1.135	0.530–2.434
	medical professionals	0.019	2.416	1.156–5.051
religion	NO	Reference		
	Yes	0.032	1.444	1.032–2.019
Frequency of receiving information regarding COVID-19	Often	0.014		
	Rarely	0.979	0.995	0.674–1.467
	Sometimes	0.007	0.704	0.546–0.909
COVID-19 Information from News App	No	Reference		
	Yes	0.027	0.741	0.568–0.966
Difficulty accessing medical treatment	Not sick or not difficult	Reference		
	Yes	0.010	1.675	1.132–2.478
Fear induced by information relevant to COVID-19	M ± SD (4.01 ± 2.64)	0.005	1.090	1.027–1.157
Desperation induced by information relevant to COVID-19	Mean ± SD(1.91 ± 1.77)	0.000	1.113	1.049–1.182
Confusion induced by information relevant to COVID-19	Mean ± SD (3.55 ± 2.65)	0.000	1.117	1.057–1.181
Sadness induced by information relevant to COVID-19	Mean ± SD (3.86 ± 2.88)	0.005	1.078	1.023–1.136
Physical Discomfort induced by information relevant to COVID-19	Mean ± SD(1.94 ± 1.73)	0.000	1.192	1.128–1.260
Mental health disorder	None	Reference		
	Having one mental health disorder	0.000	2.459	1.846–3.277
	Comorbidities with one mental health disorders	0.000	4.398	3.152–6.138
	Comorbidities with two or more mental health disorders	0.000	6.788	4.575–10.071
Daily online time	0 h < time ≤ 3 h	Reference		
	3 h < time ≤ 5 h	0.954	0.987	0.637–1.530
	5 h < time ≤ 7 h	0.568	1.140	0.727–1.790
	7 h < time ≤ 9 h	0.050	1.594	1.000–2.541
	> 9 h	0.001	2.131	1.380–3.291
Experience with traumatic incidents	Negative	Reference		
	Positive	0.000	0.434	0.312–0.605
	Did not experiences	0.000	0.492	0.370–0.654
Constant		0.000	0.004	

The multivariate logistic regression analysis controlling for the simultaneous association of risk factors revealed that fear induced by social media (OR = 1.090, CI = 1.027–1.157, *p* = 0.005), desperation induced by social media (OR = 1.113, CI = 1.027–1.157, *p* < 0.000), confusion induced by social media (OR = 1.117, CI = 1.057–1.181, *p <* 0.000), sadness induced by social media (OR = 1.078, CI = 1.023–1.136, *p =* 0.005), physical discomfort induced by social media (OR = 1.192 per score, CI =1.128–1.260, *p <* 0.000),

male gender (OR = 1.359, CI =1.054–1.752, *p* = 0.018), and other related factors were significant in the multivariate logistic regression model were summarized Table 3, thus indicating their relevance as important related factors for anxiety among the population.

## Discussion

The current study shown that 12% (736/6130) of the participants experienced moderate or severe symptoms of depression, and 7.1% (437/6130) showed moderate or severe symptoms of anxiety. Another systematic review found a wide range of anxiety and depression prevalence rates in the public during the COVID-19 pandemic, ranging from 2.7% to more than 50% [7]. These differences may derive from differences in sampling methods, assessment instrument, severity rating criteria, time point of questionnaire evaluation, and subject inclusion criteria.

Our data suggested that some demographic variables were positively associated with psychological distress. To be specific, firstly, the occupational factors could predict higher distress level, which was reflected by that medical personnel were presented more anxiety and depression symptoms than other occupations. This result was in accordance with previous research which found that medical workers were more vulnerable to facing enormous pressure, including a high risk of infection, helplessness, discrimination, isolation, burnout, lack of social support, frustration, and limited access to psychological support [18]. A lot of scholars have indicated that medical staff working in highly stressful environments were at risk of developing psychological disturbances, which suggests that medical professionals require greater access to mental health services [4, 5]. Secondly, religious belief was associated with severe/pervasive typologies of anxiety. Some researchers found that compare to non-religious individuals, religious individuals may have a generally pessimistic explanatory style (perceiving negative events as punishment), more psychological symptoms and poorer health [19]. Regarding the issues of vulnerability, people with mental illness and people with chronic physical illness who need regular medical visits are more vulnerable to on-going life events. In the context of pandemic outbreak, individuals with chronic physical illness have altered perceptions of personal control and perceived self-efficacy, which weakens their ability to cope with the stress of the COVID-19 outbreak [20]. This finding is consistent with prior studies and suggests that adults with a history of mental health disorders are more vulnerable to developing depressive and anxiety symptoms in the times of the Covid-19 crisis [20, 21]. Previous research claimed that females had higher depression and anxiety level than male during the pandemic of COVID-19 [1, 20]. However, there was also a study stated that gender was not correlated to anxiety and depression during the COVID-19 pandemic [22]. In contrast to the previous study, the current study revealed that male gender was associated with higher self-reported anxiety and depression symptoms. A systematic review disagrees with “anatomy is destiny”, instead, they suggest that different gender tend to be vulnerable to some specific social factors [23]. The gender differences from our current study may in turn reflect that the male participants tend to be more vulnerable to the stresses induced by COVID-19 due to males may undergo more financial pressure in China in terms of supporting their families during the pandemic of COVID-19.

Based on the vulnerability stress model, it claimed that the way individuals see themselves and the world seems to make a major difference to their level of vulnerability to stress [3]. Interestingly, an individual’s perception of the risk level of post-COVID-19 sequelae were found to increase the odds of having a high level of depression, which indicates that the thinking style is related to depression symptoms in the context of the COVID-19 outbreak [15]. The current findings further confirm that an individual with post-traumatic growth experiences is more resilient to current stressful events that can prevent anxiety and depression [24, 25]. These findings were in line with “inoculation hypothesis,” which suggests that experienced with a particular traumatic event may help individuals acquire skills or knowledge related to trauma and may engender a sense of competence in facing similar life crises in the future [26, 27]. This implies that it is important to help the population to obtain more positive experiences from the current stress of COVID-19 outbreak, thereby making the population more resilient rather than vulnerable.

People who mobilize effective coping skills appeared to deal with stress better than those who do not. The current study also stated that individuals who actively search for information are less likely to suffer depression, which implies that it is important to be active rather than passive during the COVID-19 outbreak. This finding was in accordance with a previous study which stated that being passive is a symptom of trauma and advocated that individuals be active in their lives, enabling them to actively cope with the difficulties during the COVID-19 outbreak to prevent psychosocial disturbances [28, 29]. The findings revealed that individuals who spend more time online are at a higher risk of depression and anxiety than individuals who browse the internet for less than 3 h.

According to stress-vulnerability model, stress generated from household, neighborhood, social media or any other environment source is more likely to make impact on mental health of individuals [3]. According to our findings, Individuals who obtain information from official media are less likely to suffer depression. Our current research discovered that the voices from medical experts and official TV programmers with concrete scientific facts and research findings may promote confidence and hope among the public rather than creating uncertainty and confusion, which may prevent people from feeling anxious and depressed. Consistent with previous research, we also found that exposure to information induced by confusion, desperation, sadness, and even somatic discomfort was linked to potentially deleterious outcomes – specifically, anxiety and depression – among the general population [30]. More specifically, information induced by fear also predisposes individuals to anxiety symptoms. Our findings indicate an urgency for health authorities to monitor health information for a more effective dissemination of information related to the COVID-19 pandemic to satisfy the demand of health information for the general public [31, 32].

### Limitations

This study had some limitations. First, this was a cross-sectional online study, and thus, any generalization of the results should be interpreted with caution. Second, we acknowledge that the current study used single items to evaluate some psychological constructs, such as negative feelings toward the information they received, and one item asked the respondent to report the number of confirmed infections in their city (0–9, 10–30, 31–100, 101–399, 400 or above. Such single-item measures are not as reliable as full scales. Third, we obtained our sample from an online survey; this may have limited the generation of the study respondents, thus leading to selection bias and exclude certain groups that have no access to the Internet (e.g. villagers) [33] Therefore, these measures should be refined, and caution should be taken in the interpretation of the findings. Fourth, the participants came from different provinces, and the unbalanced geographical distribution of participants in China could leading to selection bias. Fifth, Lack of following up makes the current research cannot make causal inferences. Last, the current study using non-COVID-19 related tools to evaluate their experiences regarding their experiences toward COVID-19 may not accurately estimate their experiences which are influenced by COVID-19. However, the study provides an understanding of the prevalence and determinants of anxiety and depression among the population in the context of the COVID-19 outbreak.

## Conclusion

Some specific individual characteristics maybe more vulnerable for anxiety and depression in the context of COVID-19 outbreak, therefore special attention should be given to individuals with these characteristics, such as individuals with mental illness or traumatic experiences, as well as to medical professionals. They should be provided with effective medical and psychological aids to increase their coping capability in the midst of the pandemic outbreak. In addition, the administration department of media should regulate the dissemination of information related to the pandemic to provide the public with a professional and reliable source of information. It is important to reduce the exposure of susceptible populations to a large amount of negative information and to maintain the mental health of the public in the context of the pandemic.

### Suggestions for future directions

It is suggested that a unified and official approach is needed to allow the public to obtain accurate information about the COVID-19 outbreak pandemic in a timely manner rather than being influenced by some controversial or confusing information from some media. Moreover, it is necessary to provide effective psychosocial support to medical workers as soon as possible to reduce their occupational burnout. In addition, it is important to provide to the public with effective access to routine medical care. Special attention and mental health services should be provided to individuals with chronic diseases and clients with mental health problems, as the COVID-19 outbreak makes them more vulnerable to mental health disorders. Last, as we work together in the face of human suffering and trauma, helping people in trouble to grow instead of being trapped in trouble could increase their ability to cope with setbacks and to grow from disasters, which is necessary in a complex and changing world.

## Supplementary information

**Additional file 1.**

## Data Availability

The data that support the findings of this study are available from the corresponding author upon reasonable request.

## Abbreviations

COVID-19: Coronavirus Disease 2019
GAD-7: Generalized Anxiety Disorder 7-item scale
PHQ-9: Patient Health Questionnaire 9-item scale
