# Supplementary material for: The psychological burden experienced by Chinese citizens during the COVID-19 outbreak: prevalence and determinants
Source: BMC Public Health. 2020 Oct 27;20:1617. doi: 10.1186/s12889-020-09723-0 (PMC7588950; doi:10.1186/s12889-020-09723-0)
Supplement: Supplementary file 1 — Additional file 1. [file 12889_2020_9723_MOESM1_ESM.docx]

**Questionnaires**

1.Gender

A：Men

B: Women

2：Age

A:16< Age ≤20

B:21< Age ≤30

C:31< Age ≤40

D:41< Age ≤50

E: Above 51

3: Career

A: Student

B: Civil servant

C: Middle and senior manager

D: blue-and-white collar workers

E: Professional staff

F: medical professionals

4: What is your monthly income？ (RMB)

A: Below 1000Yuan

B:1001-3000 Yuan

C:3001-5000 Yuan

D:5001-7000 Yuan

E:7001-10000 Yuan

F:10001 RMB Yuan

5: What is your current marital status？

A: Single

B: Married or cohabit

C: Divorced

D: Widowed

E: Others (name it)

6: Do you have any chronic physical disease that requires regular medical attention ?

A: No

B:Yes

7: Do you have religious belief?

A: No

B: Yes

8: are you national ethnic minority?

A: No

B: Yes

9: What's your educational background?

A: Middle school and below

B: Associate degree

C: university

D: degree or above

10: What is the average amount of time you spend online daily?

A: 0hour < time ≤3 hours

B: 3 hours < time ≤5 hours

C: 5 hours< time ≤7 hours

D: 7 hours < time ≤ 9 hours

E: > 9 hours

11: How often do you receive new information about the coronavirus each day?

A: Rarely

B: Sometimes

C: often

D: All the time

12: Do you have difficulty in accessing medical treatment?

A: Not sick or not difficult

B: Yes

13: Have you experienced any significant life events (natural and man-made diseases, etc.)? To what extent have they affected you ?

A: Negative

B: Positive

C: Did not experiences

14: Please select the disease (mental health disorder) you have been diagnosed from the list below

A: None

B: depression

C: anxiety

D: bipolar disorder

E. [Schizophrenia](javascript:;)

F. obsessive disorder

G. psychosomatic disorder

H. Others (name it)

15: The number of confirmed infections in their city?

A: 0-9

B: 10-30

C: 31-100

D: 101-300

E: 301 above

16: Whether they received information from any of the following channels -COVID-19 information from newspaper

A: No

B: Yes

17: Whether they received information from any of the following channels COVID-19 Information from News App

A: No

B: Yes

18: Whether they received information from any of the following channels-Self-searching COVID-19 information

A: No

B: Yes

19: Whether they received information from any of the following channels- COVID-19 information from WeChat group

A: No

B: Yes

20: Whether they received information from any of the following channels -COVID-19 information from state media on TV

A: No

B: Yes

21: Whether they received information from any of the following channels -COVID-19 information from friends

A: No

B: Yes

22: Whether they received information from any of the following channels -Message from family

A: No

B: Yes

23: Whether they received information from any of the following channels -Publicity from community worker

A: No

B: Yes

24: The extent feeling of desperation induced by information relevant to COVID-19.

(1 (not at all) to 10 (very strong))

1,2,3,4,5,6,7,8,9,10,

25: The extent feeling of fear induced by information relevant to COVID-19.

(1 (not at all) to 10 (very strong))

1,2,3,4,5,6,7,8,9,10,

25：The extent feeling of confusion induced by information relevant to COVID-19.

(1 (not at all) to 10 (very strong))

1,2,3,4,5,6,7,8,9,10,

26：The extent feeling of anger induced by information relevant to COVID-19.

(1 (not at all) to 10 (very strong))

1,2,3,4,5,6,7,8,9,10,

27：The extent feeling of sadness induced by information relevant to COVID-19. (1 (not at all) to 10 (very strong))

1,2,3,4,5,6,7,8,9,10,

28：The extent feeling of somatic discomfort induced by information relevant to COVID-19. (1 (not at all) to 10 (very strong))

1,2,3,4,5,6,7,8,9,10,

29: Each participant responded to the following items on a scale from 1 (very low chance) to 10 (very high chances). The likelihood that oneself get infected.

1,2,3,4,5,6,7,8,9,10,

30: Each participant responded to the following items on a scale from 1 (very low chance) to 10 (very high chances). The likelihood that your family member get infected?

1,2,3,4,5,6,7,8,9,10,

31: Each participant responded to the following items on a scale from 1 (very low chance) to 10 (very high chances). The likelihood of having sequela after cured?

1,2,3,4,5,6,7,8,9,10,

32: Each participant responded to the following items on a scale from 1 (very low chance) to 10 (very high chances). The likelihood that this disease will be cured?

1,2,3,4,5,6,7,8,9,10,

33. PHQ-9 Scale

34. GAD-7 Scale
